# Polygenic Risk Predicts Obesity in Both White and Black Young Adults

**DOI:** 10.1371/journal.pone.0101596

**Published:** 2014-07-03

**Authors:** Benjamin W. Domingue, Daniel W. Belsky, Kathleen Mullan Harris, Andrew Smolen, Matthew B. McQueen, Jason D. Boardman

**Affiliations:** 1 Institute of Behavioral Science, University of Colorado Boulder, Boulder, CO, United States of America; 2 Center for the Study of Aging and Human Development, Duke University Medical Center, Durham, NC, United States of America; 3 Sociology Department and the Carolina Population Center, University of North Carolina at Chapel Hill, Chapel Hill, NC, United States of America; 4 Institute for Behavioral Genetics, University of Colorado Boulder, Boulder, CO, United States of America; 5 Department of Integrative Physiology, University of Colorado Boulder, Boulder, CO, United States of America; Wayne State University, United States of America

## Abstract

**Objective:**

To test transethnic replication of a genetic risk score for obesity in white and black young adults using a national sample with longitudinal data.

**Design and Methods:**

A prospective longitudinal study using the National Longitudinal Study of Adolescent Health Sibling Pairs (n = 1,303). Obesity phenotypes were measured from anthropometric assessments when study members were aged 18–26 and again when they were 24–32. Genetic risk scores were computed based on published genome-wide association study discoveries for obesity. Analyses tested genetic associations with body-mass index (BMI), waist-height ratio, obesity, and change in BMI over time.

**Results:**

White and black young adults with higher genetic risk scores had higher BMI and waist-height ratio and were more likely to be obese compared to lower genetic risk age-peers. Sibling analyses revealed that the genetic risk score was predictive of BMI net of risk factors shared by siblings. In white young adults only, higher genetic risk predicted increased risk of becoming obese during the study period. In black young adults, genetic risk scores constructed using loci identified in European and African American samples had similar predictive power.

**Conclusion:**

Cumulative information across the human genome can be used to characterize individual level risk for obesity. Measured genetic risk accounts for only a small amount of total variation in BMI among white and black young adults. Future research is needed to identify modifiable environmental exposures that amplify or mitigate genetic risk for elevated BMI.

## Introduction

Genome-wide association studies (GWAS) have discovered genetic loci that associate with obesity risk [1]. Genetic risks manifest early in life; children at higher genetic risk gain weight more rapidly during infancy and early childhood and reach adiposity rebound earlier in life and at higher body-mass-index (BMI) [2]–[4]. In turn, this rapid growth early in life functions as a mediator of genetic risk for adult obesity [4]. These observations suggest the possibility that genetic information can inform research to understand pathogenesis of obesity in childhood, with the goal of improving prevention and treatment [5], [6].

Single-nucleotide polymorphisms (SNPs) discovered in GWAS of obesity phenotypes have small effects; the most penetrant SNP predicts an increase of less than one half of one BMI point in adult samples [7]. As a result, many samples designed to investigate obesity etiology are underpowered to study individual GWAS discoveries. Combining information on multiple GWAS discovered SNPs to compute a “genetic risk score” can provide a tool for investigating genetic contributions to obesity etiology in samples far smaller than are needed for GWAS [8], [9].

To date, most genetic risk score research on obesity has focused on European-descent samples [2]–[4], [10], [11]. Expanding the scope of genetic research to consider other populations is a public health priority [12]. A challenge is that GWAS-discovered SNPs may not cause disease themselves, but may instead serve as proxy measures of causal variants elsewhere in the DNA sequence. Allele frequencies and patterns of linkage disequilibrium vary across racial and ethnic groups [13]. One implication of these differences is that a SNP measured in a GWAS may serve as a proxy for a given causal variant in the GWAS discovery population, but not in a new sample drawn from a different ethnic group. This problem is compounded when GWAS discoveries are followed up in existing databases that may not contain the original GWAS-discovered SNP and proxies must be selected [14].

An increasing number of large and representative samples of adults from diverse populations are available with genome wide data from respondents. Genetic risk scores are a promising tool for population health research using such datasets but relatively small differences in allele frequency and LD patterns across groups may complicate the interpretation of genetic risk associations [15], [16]. A necessary first step is to test scores in different ethnic populations and establish whether a genetic risk score based on discoveries made in one population will translate to another.

This study tests transethnic replication of a genetic risk score for obesity in white and black young adults in the National Longitudinal Study of Adolescent Health (Add Health) Sibling Pairs Study. We tested genetic associations with obesity at two waves (when respondents were roughly 18–26 and 24–32 years old, respectively). We next tested genetic associations with change in obesity over the 7-year interval between waves. We conducted tests separately in the white and black samples and then tested for differences in genetic associations between the two groups. We also compared the performance of the GRSs computed from SNPs identified in samples of European ancestry to additional GRSs that incorporate SNPs discovered in a recent GWAS of African Americans.

## Materials and Methods

### Sample

Add Health is a nationally representative cohort (*n* = 20,745, aged 12–20 years at Wave 1 in 1994–95) drawn from a probability sample of 80 US high schools and 52 US middle schools, representative of US schools in 1994–95 with respect to region, urban setting, school size, school type and race or ethnic background. The Wave 3 (2001–2002) and 4 (2008–2009) data collection originally contained *n* = 15,197 individuals (then aged 18–26 years, mean age 22.3 years) and *n* = 15,701 individuals (then aged 24–32 years, mean age 28.9 years) respectively. The Add Health Sibling Pairs [17] data used here consists of 1,595 individuals (58% white, 42% black) from 965 families (564 sibling pairs, 30 sibling trios, 2 sibling quads, and 369 singletons) who were genotyped from samples collected during Wave 4 of the Add Health study (this phase of the Add Health study genotyped only the Sibling Pairs). The Sibling Pairs cohort oversampled black respondents (42.1% of Sibling Pairs as compared to 28.4% of all Add Health are black). The Sibling Pairs cohort did not differ from the full Add Health sample in terms of gender, age, maternal education, or health of the respondents (detailed results available upon request). The Siblings Pairs cohort was roughly 0.25 BMI units above the full Add Health sample at both Waves 3 and 4 but the waist to height ratios were identical in both groups. Missing data for phenotypic information reduced the number of respondents that could be used in the below analyses. The exact reduction in sample varied by phenotype, but the minimum white sample used in analysis was 773 respondents and the minimum black sample was 530 respondents (n = 1,303).

### Genetic Risk Score

Genotyping was conducted with the Illumina HumanOmni1-Quad v1 platform using DNA extracted (via Oragene saliva collection) from 1,946 individuals at Wave 4. After quality controls (see http://ibs.colorado.edu/jb/pairsgwasqc.pdf), the genetic database included 1,886 individuals with valid data on 940,862 single nucleotide polymorphisms. Our analysis focused on non-Hispanic white and black individuals as indicated by self-report (n = 1,303) and SNPs with missing call rates below 5% (this criteria resulted in the removal of 18,665 SNPs from the original set of 959,862 SNPs). Principal components, which are commonly used to adjust for population stratification in GWAS [13], were computed with 231, 649 SNPs (selected from the full set of SNPs to be in linkage equilibria) from chromosomes 1–22.

We constructed three multi-locus indicators of genetic risk for obesity. The genetic risk score for European-descent populations (GRS-E) included 31 SNPs discovered in GWAS of adult BMI in European-descent individuals [7] (this risk score is available through the restricted use mechanism of Add Health). The genetic risk score for African-Americans (GRS-A) included 8 SNPs discovered in GWAS of adult BMI in African American and African individuals [18]. Genetic risk scores were created from sets of SNPs identified as genome-wide significant in their respective studies. We constructed a third genetic risk score (GRS-Omni) from the complete set of loci discovered in either GWAS. For loci in or near the genes *FTO*, *SEC16B*, and *GNPDA2*, the two GWAS identified loci in high linkage disequilibrium (r>0.9) and a single tag SNP was selected. The method used to compute the risk scores was the same for each set of SNPs. We summed the BMI-increasing alleles (as identified in each GWAS) for each SNP and then summed these counts of BMI-increasing alleles across the SNPs. Due to the lack of a comparable effect size metric between the Speliotes et al. [7] and Monda et al. [18], we use unweighted risk scores in most analyses although we also report weighted risk scores for GRS-E (using weights from [7]) to examine sensitivity to the weighting. For individuals with missing information on SNPs to be included in a risk score (8% of individuals were missing information on at least one SNP in GRS-Omni but no individual was missing information on more than four SNPs), we calculated pro-rated genetic risk scores by dividing the calculated genetic risk score by the number of SNPs with available calls and multiplying by the total number of SNPs in the score.

The SNPs included in the genetic risk scores are reported in **Tables 1** and **2**. Since base rates of the risk alleles varied between the white and black samples, we standardized the weighted sums of risk alleles to have a mean of 0 and a SD of 1 separately in each of the white and black samples for each genetic risk score. (For analyses conducted using both white and black samples, results were consistent if risk scores are instead standardized across race). In the black sample, GRS-E and GRS-Omni were highly correlated (r = 0.91); GRS-A was less correlated with both (with GRS-E, r = 0.23; with GRS-Omni, r = 0.54). Effect-sizes reported from genetic risk score analyses reflect the effect of a one standard-deviation increase in genetic risk on obesity outcomes.

**Table 1:** Single-nucleotide polymorphisms included in the genetic risk score for Europeans (GRS-E).

Chr	Nearest Gene	Speliotes et al. [7] SNP	Add Health SNP	R2 with GWAS SNP*	Risk Allele	Other	White MAF	Black MAF	Weight [7]
1	*PTBP2*	rs1555543	rs10489741	1	a	g	0.46	0.41	0.06
	*TNNI3K*	rs1514175	rs1514175	1	a	g	0.43	0.35	0.07
	*NEGR1*	rs2815752	rs2815752	1	a	g	0.37	0.46	0.13
	*SEC16B*	rs543874	rs543874	1	g	a	0.20	0.26	0.22
2	*LRP1B*	rs2890652	rs1523702	0.702	c	t	0.13	0.06	0.09
	*FANCL*	rs887912	rs2192497	1	c	t	0.26	0.09	0.10
	*TMEM18*	rs2867125	rs2867125	1	c	t	0.16	0.11	0.31
	*RBJ*	rs713586	rs713587	0.967	t	c	0.47	0.16	0.14
3	*ETV5*	rs9816226	rs7635103	0.618	a	c	0.27	0.47	0.14
	*CADM2*	rs13078807	rs9852127	1	a	g	0.25	0.06	0.10
4	*GNPDA2*	rs10938397	rs10938397	1	g	a	0.43	0.20	0.18
	*SLC39A8*	rs13107325	rs13107325	1	t	c	0.13	0.02	0.19
5	*FLJ35779*	rs2112347	rs10057967	1	t	c	0.39	0.48	0.10
	*ZNF608*	rs4836133	rs4836133	1	a	c	0.48	0.22	0.07
6	*NUDT3*	rs206936	rs206936	1	g	a	0.21	0.48	0.06
	*TFAP2B*	rs987237	rs987237	1	g	a	0.16	0.09	0.13
9	*LRRN6C*	rs10968576	rs10968576	1	g	a	0.30	0.17	0.11
11	*RPL27A*	rs4929949	rs11041994	0.966	c	a	0.46	0.47	0.06
	*BDNF*	rs10767664	rs7103411	1	t	c	0.15	0.07	0.19
	*MTCH2*	rs3817334	rs7124681	1	a	c	0.39	0.26	0.06
12	*FAIM2*	rs7138803	rs7138803	1	a	g	0.36	0.16	0.12
13	*MTIF3*	rs4771122	rs9512699	0.874	g	a	0.19	0.13	0.09
14	*NRXN3*	rs10150332	rs17109256	1	a	g	0.19	0.23	0.13
15	*MAP2K5*	rs2241423	rs2241423	1	g	a	0.27	0.38	0.13
16	*GPRC5B*	rs12444979	rs12444979	1	c	t	0.14	0.08	0.17
	*FTO*	rs1558902	rs1421085	1	c	t	0.42	0.08	0.39
	*SH2B1*	rs7359397	rs3888190	0.965	a	c	0.44	0.27	0.15
18	*MC4R*	rs571312	rs571312	1	a	c	0.16	0.37	0.23
19	*QPCTL*	rs2287019	rs2287019	1	c	t	0.21	0.10	0.15
	*KCTD15*	rs29941	rs29942	1	g	a	0.29	0.15	0.06
	*TMEM160*	rs3810291	rs3810291	1	a	g	0.35	0.18	0.09

Note: SNPs are the set of genome-wide significant SNPs discovered in the GWAS meta-analysis by the GIANT Consortium [7]. Weights are the effect-sizes estimated in that analysis. In cases where the original GWAS SNP was not available in the Add Health genotype database, linkage proxies were identified using the Broad Institute’s SNAP tool [35] (1000 Genomes (Pilot 1) CEU reference sample). No proxy was available for rs11847697 near *PRKD1*. Alleles are reported according to dbSNP. Frequencies of BMI-increasing alleles are reported separately for white and black Add Health study members meeting genotype quality control criteria.

**Table 2:** Single nucleotide polymorphisms (SNPs) included in the African American genetic risk score (GRS-A).

Chr	Nearest Gene	Monda et al. [18] SNP	Add Health SNP	R2 with GWAS SNP*	Risk Allele	Other	White MAF	Black MAF
1	*SEC16B*	rs543874	rs543874	1	G	A	0.2019	0.0571
2	*ADCY3*	rs7586879	rs6752483	0.849	T	C	0.4903	0.042
4	*GNPDA2*	rs348495	rs10938397	0.698	G	A	0.4307	0.048
5	*GALNT10*	rs7708584	rs7719067	0.836	A	G	0.4471	0.05
6	*KLHL32*	rs974417	rs974417	1	G	T	0.1154	0.04
7	*MIR148A-NFE2L3*	rs10261878	rs1966841	0.961	G	A	0.399	0.03
16	*FTO*	rs17817964	rs3751812	0.707	T	C	0.399	0.074
18	*MC4R*	rs6567160	rs6567160	1	C	T	0.1635	0.062

Note: SNPs are the set of genome-wide significant SNPs discovered in the GWAS meta-analysis by Monda and colleagues [17]. In cases where the original GWAS SNP was not available in the Add Health genotype database, linkage proxies were identified using the Broad Institute’s SNAP tool [35] (1000 Genomes (Pilot 1) YRI reference sample). Frequencies of BMI-increasing alleles are reported separately for white and black Add Health study members meeting genotype quality control criteria.

### Anthropometry

Anthropometric assessments of the Sibling Pairs were conducted at Add Health waves 3 and 4. Weight and height were measured during in-person interviews [19]. Participants were weighed without shoes on a digital bathroom scale (to the nearest half-pound in Wave 3 and tenth of a kilogram in Wave 4). The scales had a maximum of 330 pounds (200 kg); individuals above these thresholds were coded as being at the maximum scale weights (9 and 19 individuals were coded at this maximum weight for Waves 3 and 4 respectively). Heights were measured to the nearest 1/8^th^ of an inch. BMI was computed as kilograms per height in meters squared. Obesity was defined as BMI≥30. Anthropometric characteristics of the white and black samples are described in **Table 3**.

**Table 3:** Characteristics of white and black young adults in the Add Health Sibling Pairs sample.

	Whites (N = 918)		Blacks (N = 677)		p-value for difference
	Mean	SD	Mean	SD	
% Male	0.48	0.50	0.46	0.50	0.44
BMI-Wave 3	25.78	5.80	26.39	6.32	0.07
BMI-Wave 4	27.86	6.60	29.34	7.44	0.00
BMI Change	2.10	3.93	2.69	3.99	0.01
Waist/Height-Wave 4	0.57	0.10	0.58	0.11	0.15
% Obese-Wave 3	0.22	0.42	0.26	0.44	0.13
% Obese-Wave 4	0.33	0.47	0.40	0.49	0.01

Note: Data are for the Sibling Pairs of the National Longitudinal Study of Adolescent Health [17].

### Statistical Analysis

We tested genetic associations with BMI and obesity using linear and logistic regression models, respectively. Analyses were conducted separately in whites and blacks. We analyzed change in BMI and obesity from Wave 3 to Wave 4 by including the level of Wave 3 BMI (or obesity) as a predictor in multivariate regression models predicting Wave 4 BMI (or obesity). All regressions were estimated using multilevel models (random intercept) to account for the non-independence of observations within families [20] and were adjusted for age and sex. All continuous outcomes were standardized within race. There was greater variability in the BMI of black respondents, see **Table 3**. Effect sizes reflect the effect in SDs of a 1 SD increase in genetic risk score on BMI or BMI change or on the log odds of obesity or in log odds of change in obesity (reported as odds ratios).

We conducted two additional sets of analyses. First, because a previous study reported that the predictive performance of an obesity genetic risk score differed in black and white populations [4], we tested for differences in genetic associations with obesity phenotypes between blacks and whites. These analyses combined black and white respondents were into a single dataset. The models included a main effect term for race, a main effect term for genetic risk, and an interaction term testing race differences in the magnitude of the genetic effect.

Second, to rule out confounding by unmeasured population stratification [21], we conducted a sibling difference analysis using family fixed effects. The sibling difference analysis tested whether, within a pair of siblings who grew up in the same household, the sibling with the higher genetic risk score had the higher BMI. Sibling difference analyses provide a control for any unmeasured population stratification [22]. To maximize statistical power for these models, we analyzed all available data from waves 3 and 4 and introduced an individual-level random intercept to account for the non-independence of observations within individuals.

## Results

White and black young adults at higher genetic risk as measured by GRS-E had higher BMIs compared to their lower genetic risk age-peers (**Table 4**). For whites, genetic associations with BMI were 0.16 at Wave 3 and 0.17 at Wave 4 (p<0.001 for both; results with unweighted risk scores are reported unless indicated otherwise). For blacks, genetic associations with BMI were r = 0.14 at Wave 3 and r = 0.13 at Wave 4 (p<0.01 for both). Genetic associations with BMI did not differ between the white and black samples at either Wave (p>0.75 for both tests).

**Table 4:** Genetic associations with body-mass index and obesity in white and black young adults in the Add Health Sibling Pairs sample estimated using the genetic risk score for Europeans (GRS-E).

	Obesity Phenotype	White Sample		Black Sample		*p-value for difference*
Unweighted		B [95% CI]				
	BMI-Wave 3	0.16***	[0.09, 0.23]	0.14**	[0.06, 0.23]	*0.96*
	BMI-Wave 4	0.17***	[0.10, 0.24]	0.13**	[0.04, 0.21]	*0.76*
	Change	0.06*	[0.01, 0.10]	0.01	[−0.04, 0.05]	*0.23*
		OR [95% CI]				
	Obesity-Wave 3	1.42**	[1.14, 1.78]	1.19	[0.96, 1.48]	*0.38*
	Obesity-Wave 4	1.54***	[1.30, 1.83]	1.19	[0.98, 1.45]	*0.06*
	Change	1.43**	[1.14, 1.79]	1.09	[0.83, 1.45]	*0.12*
Weighted		B [95% CI]				
	BMI-Wave 3	0.16***	[0.09, 0.23]	0.16***	[0.07, 0.24]	*0.83*
	BMI-Wave 4	0.18***	[0.10, 0.25]	0.14***	[0.06, 0.22]	*0.85*
	Change	0.06**	[0.02, 0.11]	0.01	[−0.03, 0.06]	*0.21*
		OR [95% CI]				
	Obesity-Wave 3	1.37**	[1.10, 1.71]	1.25*	[1.01, 1.56]	*0.68*
	Obesity-Wave 4	1.56***	[1.31, 1.85]	1.22*	[1.00, 1.48]	*0.05*
	Change	1.48***	[1.18, 1.86]	1.10	[0.83, 1.46]	*0.07*

* p<.05; ** p<.01; *** p<.001.

Note: All data come from the National Longitudinal Study of Adolescent Health Sibling Pairs [17]. Genetic risk was measured using the genetic risk score for Europeans (GRS-E). Regressions were estimated using multi-level models [20] to account for the clustering of observations within families and adjusted for age and sex. Change models were estimated by including Wave 3 outcomes as covariates in regression models predicting Wave 4 outcomes.

White and black young adults at higher genetic risk as measured by GRS-E were also more likely to be obese compared to those at lower genetic risk. For whites, genetic associations with obesity were OR = 1.42 [1.14–1.78] at Wave 3 and OR = 1.54 [1.30–1.83] at Wave 4. For blacks, genetic associations with obesity were OR = 1.19 [0.96–1.48] at Wave 3 and OR = 1.19 [0.98–1.45] at Wave 4. Genetic associations with obesity were similar in blacks and whites at Wave 3 (p = 0.38). At Wave 4, the effect magnitude was larger among whites as compared to blacks (p = 0.06). Over the 7-year interval between waves 3 and 4, white young adults at higher genetic risk gained more weight and were more likely to become obese as compared to their lower genetic risk age-peers (for BMI, r = 0.06, p<0.05; for obesity status, OR = 1.43 [1.14–1.79]). Genetic risk was not associated with change in BMI and obesity among blacks. Insufficient statistical power is a possible, but unlikely, explanation for the failure to detect an association between the GRS and BMI change in the black Sibling Pairs; based on the effect observed in the white Sibling Pairs, we have 73% power in the black Sibling Pairs sample. However, the Add Health Sibling Pairs is slightly underpowered as a dataset to test black-white differences in genetic risk score associations with BMI change; power for these analyses was below 50%.

Weighting GRS SNPs by the effect-sizes estimated in GWAS had a modest effect on genetic risk score performance. Because weights customize the contribution of each SNP to the GRS according to its effect on BMI, the expectation is that a weighted GRS will provide superior prediction as compared to a GRS in which all SNPs are weighted equally. For most of the phenotypes analyzed, test-statistics and effect sizes were similar for weighted and un-weighted scores (**Table 4**).

We also analyzed genetic associations with a more direct measure of adiposity: the ratio of waist-circumference to height [23]–[25]. Similar to results for BMI, white and black young adults at higher genetic risk as measured by GRS-E had higher waist-height ratios as compared to their lower genetic risk peers (for whites r = 0.16, for blacks r = 0.13; p<0.001 for whites and p<0.01 for blacks; as with BMI, the waist-height ratio was standardized in each racial group). Genetic associations did not differ between white and black samples (p = 0.86).

As a final test of the performance of GRS-E, we examined sibling differences in BMI using fixed effects regression techniques. Because previous race-stratified analyses yielded parallel results for whites and blacks with BMI, we pooled samples for the sibling differences analysis (for added stringency, we also included the first four principal components). Results were little changed from our original analyses. Across the Wave 3 and 4 assessments, the sibling with the higher genetic risk score had higher BMI (b = 0.62, p = 0.06; BMI was unstandardized in this analysis).

**Figure 1:**
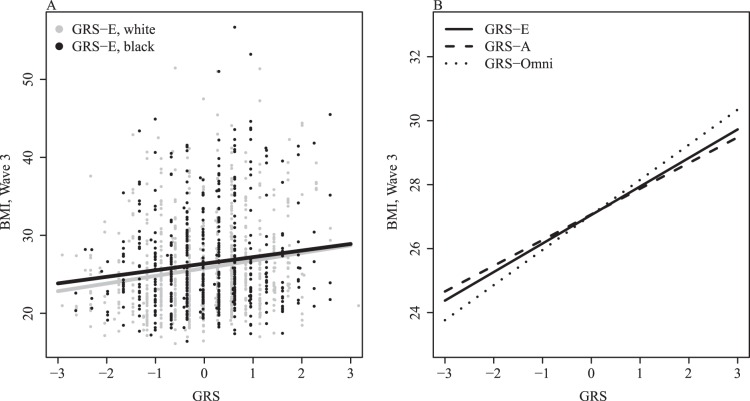
Comparison of GRS predictions. Panel A compares the predictive performance of GRS-E in both white and black samples of Add Health respondents based on a model where Wave 3 BMI is predicted by only GRS (separately in each racial group). Panel B focuses on predictions based on the three risk scores for only the black sample of respondents. The fitted lines are based on linear models controlling for age, sex, and one of the risk scores. The predictions assume an age of 21 and female.

To test whether incorporating SNPs discovered in GWAS of African-descent individuals would improve GRS performance in African Americans, we compared the performance of GRS-E to that of GRS-A and GRS-Omni in the black Add Health Sibling Pairs (n = 667). **Table 5** reports results for the analyses described above using the alternate genetic risk scores**.** For BMI, results were similar for all genetic risk scores (see also **Figure 1**). Inclusion of SNPs discovered in the Monda et al. [18] GWAS of BMI in African Americans and Africans modestly improved the performance of the genetic risk score (GRS-A and GRS-Omni scores performed better than GRS-E in the black Sibling Pairs).

**Table 5:** Genetic associations with body-mass index and obesity among black young adults in the Add Health Sibling Pairs Sample estimated using the genetic risk scores for Europeans (GRS-E), African Americans (GRS-A), and the composite genetic risk score (GRS-Omni).

Obesity Phenotype	GRS-E		GRS-A		GRS-Omni	
	B [95% CI]					
BMI-Wave 3	0.14**	[0.06, 0.23]	0.12**	[0.04, 0.20]	0.17***	[0.09, 0.26]
BMI-Wave 4	0.13**	[0.04, 0.21]	0.12**	[0.04, 0.20]	0.15***	[0.07, 0.24]
Change	0.01	[−0.04, 0.05]	0.03	[−0.02, 0.07]	0.02	[−0.03, 0.06]
	**OR [95% CI]**					
Obesity-Wave 3	1.19	[0.96, 1.48]	1.29*	[1.05, 1.59]	1.27*	[1.03, 1.58]
Obesity-Wave 4	1.19	[0.98, 1.45]	1.26*	[1.04, 1.52]	1.28*	[1.06, 1.55]
Change	1.09	[0.83, 1.45]	1.19	[0.90, 1.56]	1.18	[0.89, 1.55]

* p<.05; ** p<.01; *** p<.001.

Note: All data come from the National Longitudinal Study of Adolescent Health Sibling Pairs [17]. Regressions were estimated using multi-level models [20] to account for the clustering of observations within families and adjusted for age and sex. Change models were estimated by including Wave 3 outcomes as covariates in regression models predicting Wave 4 outcomes.

## Discussion

We used data from a prospective longitudinal study to examine the effects of cumulative genetic risk on body-mass phenotypes in white and black young adults. Consistent with findings from previous studies using samples of white adults [4], [14], [26], individuals at higher genetic risk had higher BMI, were more likely to be obese, and had higher levels of body fat (as measured by waist-height ratio) compared to their lower genetic risk peers. A novel finding of our study is that magnitudes of genetic associations, especially with BMI, were similar in white and black samples. Results for white and black samples differed in analyses of change over time. In the white sample, young adults at higher genetic risk gained more weight and were more likely to become obese as compared to those at lower genetic risk. In the black sample, these associations were in the same direction, but were smaller in magnitude and not statistically significant. We further showed that alternate genetic risk scores derived from GWAS of Europeans and from GWAS of African Americans and Africans performed similarly in predicting BMI and obesity in African American young adults. We also note that although the risk score created from the GWAS on African Americans utilized a small number of SNPs, the association is unlikely to be driven by a single SNP given the fact that the weights cited in the GWAS [18] are relatively consistent with the least predictive SNP being only half as predictive as the most predictive SNP. In contrast, the most predictive SNP in the Speliotes et al. [7] GWAS is ten times predictive as many of the other SNPs.

The magnitudes of genetic associations with obesity phenotypes were small; e.g. a one SD increase in GRS-E predicted a 0.14 SD increase in BMI at Wave 3 for those in the black sample. These translate to roughly a 0.9 point increase in BMI. Using the genetic risk score as an individual-level risk assessment would produce too many false positive and false negative results to recommend immediate clinical translation [27]. Nevertheless, the small effects we report are consistent with effect sizes for many other biomarkers routinely assessed in clinical settings [28]. Moreover, sibling comparison analyses showed that genetic associations with BMI were detectable within sibling pairs, indicating that genetic effects, although small, are apparent even in individuals who share those risk factors for obesity defined by the family environment. More research is needed to understand how GWAS-discovered genetic risks combine with other risk factors in order to understand complex traits [16].

Our findings have important implications for the use of genetic risk scores in obesity research. Our study provides evidence of transethnic replication of a genetic risk score for obesity based on GWAS discoveries in European-descent samples in a population-based black cohort. Some of the SNPs discovered in GWAS of obesity in European-descent samples have been replicated in black samples [18], [29], but transethnic replication a GWAS-based genetic risk score for obesity was uncertain. A recent analysis of data from the Atherosclerosis Risk in Communities (ARIC) study found that associations between the genetic risk score and obesity were weaker in blacks as compared to whites [14]. In that study, the genetic association with body-mass-index was r = 0.13 in whites, a little less than what we report from Add Health, but among blacks the magnitude of this association was reduced by two thirds. Research is needed to determine the cause of the discrepancy between results from the ARIC and Add Health cohorts. Three obvious differences in the cohorts are their age–ARIC participants are in their 50s and 60s whereas Add Health participants are in their 20s; the timing of assessments–the ARIC cohort was assessed in the late 1980s and 1990s whereas the Add Health cohort was assessed in the 2000s; and the geographic locations where individuals in the samples lived–black ARIC participants lived in North Carolina and Mississippi whereas Add Health participants were representatively drawn from across the United States. Thus, age, period, and cohort factors as well as factors related to place all represent candidate explanations [4], [26], [30].

Developmental processes, gene-environment correlations, and gene-environment interactions are promising targets for future inquiry into variation in the effects of obesity genetic risk scores [31]–[33]. Research in European-descent samples has identified rapid childhood growth, partly arising from decreased satiety response, as a mediator of genetic risk for obesity and points to sedentary lifestyle and poor diet as important moderators of genetic risk for obesity [10], [11], [14], [34]. These factors and others may differ between Add Health black respondents and the older African Americans examined in other studies, contributing to the small differences in genetic associations with obesity that we observe at Wave 4. Now that this study has provided evidence for transethnic replication of the genetic risk score in black young adults, future research can investigate the role of gene-environment interactions in determining genetic associations with obesity in blacks.

We acknowledge limitations. First, we provide evidence for transethic replication of genetic risk score associations with obesity phenotypes in white and black young adults, but results may not generalize to other ethnic groups. Additional studies focusing on other populations are needed. Second, the obesity phenotypes we examined were derived from anthropometric assessments that may capture body-size variation due to muscle mass as well as adiposity. We did replicate genetic associations with body-mass index using waist-circumference-to-height ratio, a superior measure of adiposity (that was available only for Wave 4). Finally, Add Health is a nationally representative sample, but the Sibling Pairs Study subsample that we analyzed is smaller and may not represent children who do not have siblings. As with all genetic research, replication of findings in additional samples is a priority.
